# Habitat modification by marram grass negatively affects recruitment of conspecifics

**DOI:** 10.1007/s00442-024-05525-y

**Published:** 2024-03-15

**Authors:** Carlijn Lammers, Annika Schmidt, Tjisse van der Heide, Valérie C. Reijers

**Affiliations:** 1 Department of Coastal Systems, Royal Netherlands Institute for Sea Research, 1790 Den Burg, AB The Netherlands; 2 Conservation Ecology Group, Groningen Institute for Evolutionary Life Sciences, University of Groningen, 9700 Groningen, AA The Netherlands; 3 Department of Physical Geography, Faculty of Geosciences, Utrecht University, 3508 Utrecht, TC The Netherlands

**Keywords:** Habitat modification, Coastal dunes, Plant recruitment, *Ammophila arenaria*, Sediment dynamics

## Abstract

Ecosystem engineers alter their environment often benefiting their own survival and growth yielding self-reinforcing feedbacks. Moreover, these habitat modifications have been found to facilitate recruitment of conspecifics for some species, while for others engineering inhibits recruitment. Whether dune grasses facilitate or inhibit recruitment of conspecifics is yet unknown. Here, we investigated how habitat modification by European marram grass (*Ammophila arenaria*) through embryonic dune development affects recruitment from seeds and marine dispersed rhizome fragments. Specifically, we tested at three locations with different dune morphologies how habitat modification affected natural seed and rhizome presence and shoot emergence from plots in which seeds or rhizome fragments were added. In addition, we investigated how sediment burial (i.e., the main effect of habitat modification by dune grasses) affected germination and emergence in a controlled experiment. Results show that regardless of habitat modification or beach width, seeds and rhizomes were absent in natural conditions. Habitat modification negatively affected shoot emergence from seeds (8 × less) and rhizomes (4 × less) and was negatively related to sediment dynamics. Furthermore, fewer seedlings were found with higher elevations. In controlled laboratory conditions, the highest seedling emergence was found with slight burial (0.5-3 cm); both germination and seedling emergence decreased as seeds were buried deeper or shallower. Overall, habitat modification by marram grass negatively affects recruitment of conspecifics through increased sediment dynamics and elevation. Consequently, storm events or eradication programs that include removal of adult vegetation—which leads to an unmodified system—might benefit new recruitment from seeds or clonal fragments.

**Supplementary Information:**

The online version contains supplementary material available at 10.1007/s00442-024-05525-y.

## Introduction

The early stages in the life cycle of plants – from seed and sprout to small plants – are often the bottleneck for successful species long-term establishment thereby affecting population dynamics and distribution (Grubb 1977). Such bottlenecks arise as the environmental tolerance in early stages often differs from the adult stages that are typically more resistant (Grubb 1977; Del Vecchio et al. 2020). For example, American beach grass (*Ammophila breviligulata*) is vulnerable to desiccation in its seedling stage but drought tolerant as adult (Laing 1958; Maun 1994). Apart from recruitment from seeds, clonal plants have vegetative growth as a second mode of reproduction which can have different establishment requirements compared to seeds (Harris and Davy 1986; van der Putten 1990). In general, populations of clonal plants are maintained and expanding through vegetative growth, rather than through establishment from seeds. However, after disturbance events establishment via seed dispersal allow for (re)colonization of new regions at larger distances (Silvertown 2008; Herben et al. 2015). Ecosystems that experience frequent and regular physical disturbances, such as fluvial and coastal ecosystems (e.g., rivers, salt marshes and coastal dunes), are often regulated by strong bottlenecks for species establishment. Here, flooding and sediment transport by water and wind inhibits plant establishment and recruitment only occurs during relatively calm periods called ‘Windows of Opportunity’ (Corenblit et al. 2007; Balke et al. 2014).

In harsh coastal environments, ecosystem engineering plants, such as mangroves, cordgrasses and dune grasses, alter their physical environment invoking feedback loops that improve living conditions for themselves and associated species (Jones et al. 1994; Crain and Bertness 2006). Throughout an ecosystem engineering plant’s life cycle, the effect of environmental conditions changes from influencing germination and establishment potential to influencing their ability to grow, expand and engineer their environment (Balke et al. 2011; Schwarz et al. 2015). For some coastal ecosystem engineers, these habitat modifications also benefit recruitment of conspecifics. For instance, mussels and oysters provide attachment structures and predation shelter for larval recruitment and mangrove roots or seagrass shoots stabilize the soil through water flow reduction which benefits seedling survival (Zipperle et al. 2009; Balke et al. 2011; van der Heide et al. 2014; Rodriguez-Perez et al. 2019). However, habitat modification can also negatively affect new recruitment. For example, ecosystem engineering by smooth cordgrass (*Spartina alterniflora*) can result in increased soil ammonia levels, which in turn can lower seedling establishment (Lambrinos and Bando 2008). Whether habitat modification by dune grasses facilitates or inhibits recruitment of conspecifics is yet unknown.

Dune grasses are the dominant ecosystem engineering species in temperate coastal dune systems. As these systems occur at the land-sea interface, there is a sharp change in environmental conditions such as salinity levels, wind intensity, sediment transport and soil temperatures from sea inland (Hesp 1989; Martínez and Psuty 2004). Conditions close to the shoreline are generally too harsh for establishment, but above the high-water line dune grasses can establish during periods of low or absent disturbance (Balke et al. 2014). Once seedlings or plant fragments are rooted, they clonally expand and start accumulating sediment by reducing flows of wind with their physical structures, thereby forming embryonic or incipient dunes. Over time, these embryonic dunes can develop into more stable foredunes (Hesp 2002; van Puijenbroek et al. 2017a). These plant-mediated modifications to the environment lead to an increase in bed-level elevation, soil moisture levels and a drop in soil temperatures (Baldwin and Maun 1983). All these changes can potentially benefit recruitment of conspecifics, due to reduced flood risk and with increased moisture and lower temperatures desiccation is less likely, which are thought to be the main factors determining seedling survival in coastal dunes (Maun 1994). However, sediment accretion might also negatively affect recruitment as burial poses direct stress for many species (Maun and Lapierre 1986; Lim 2012; Bonte et al. 2021).

The aim of this study is to determine how habitat modification by European marram grass (*Ammophila arenaria* hereafter referred to as marram grass) affects recruitment of conspecifics. Marram grass is one of the dominant dune grasses along the Northwestern European coast. It is known for its efficient dune building and therefore has been introduced in coastal regions worldwide (e.g. Bakker 1976; Hacker et al. 2012; Hertling and Lubke 1999; Hilton 2006). In Europe, marram grass flowers between June and August, followed by a peak in seed dispersal in September (Huiskes 1979). Seeds that disperse in September will only be able to germinate the next spring since the seeds need cold stratification (Huiskes 1979). Dispersal via rhizome fragments, the second, asexual, mode of recruitment, is dependent on storm events (Hilton and Konlechner 2011). As seed or rhizome availability is the first requirement for successful establishment, we determined seed and marine dispersed rhizome availability in spring at three locations with different beach-dune morphologies. Second, we experimentally tested shoot emergence from seeds and rhizome fragments in modified and unmodified habitats at these three locations. Lastly, we examined the effect of sediment burial (i.e., the most important effect of habitat modification by marram grass) on seed germination and emergence in controlled conditions.

We hypothesize that most marram grass seeds will be dispersed close to adult vegetation (Pope 2006), and we therefore expect a higher seed availability close to vegetation. Furthermore, the presence of marine dispersed rhizomes is expected to be dependent on the presence of vegetation (as a source) in combination with beach width, with higher chances of erosion and potential rupture of adult vegetation on short, steep beaches than on wide beaches with a gentle slope (Anthony 2013; Itzkin et al. 2021). Since we expect that habitat modification can both have negative (e.g., sediment burial) and positive (e.g., increased moisture levels) effects it is hard to predict whether it will facilitate or inhibit recruitment (Baldwin and Maun 1983; Lim 2012; Bonte et al. 2021). Furthermore, we expect lower seedling emergence with increased burial in controlled conditions.

## Methods

### Field sites

**Fig. 1:**
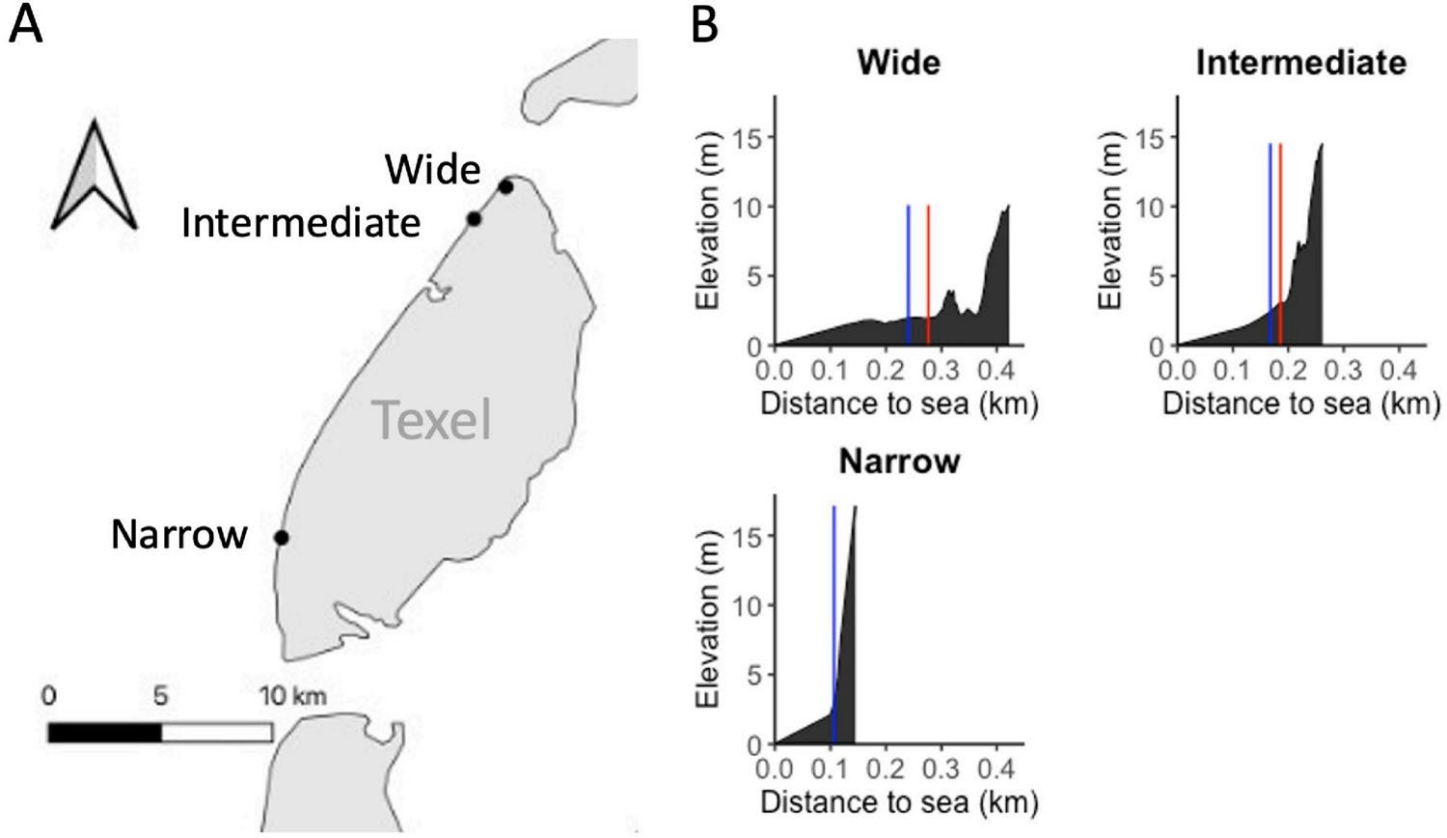
**A** Location of the field sites on Texel. **B** Dune profiles of the locations based on a transect with RTK-GPS. The blue vertical lines indicate plot locations in the unmodified zone (on the beach) and the red vertical lines the plots in the modified zone (in the embryonic dunes). At the narrow location no embryonic dunes were present

We selected three beach – dune locations on the Dutch Wadden Island Texel. The widest system was ± 420 m (mean sea level (MSL)—top foredune), the intermediate location ± 260 m and the narrow location ± 140 m wide (Fig. 1, Fig. S1). On the wide and intermediate locations embryonic dunes (i.e., a modified environment) were present, while on the narrow beach they were absent. At the wide location embryonic dunes stretched ± 170 m and at the intermediate location ± 55 m. As a consequence of beach width, presence of vegetation and other environmental conditions, dune morphologies differed between locations (Fig. 1, Fig. S1).

### Natural seed and rhizome availability

The natural presence of seeds and marine dispersed rhizomes in the early successive stages of dunes was determined at the selected locations in March 2021 by sieving sediment cores (ø10 cm, 20 cm depth) over a 1 mm mesh. The cores were placed along a transect with 3 m spacing between cores (3 parallel transects interspaced ± 4 m per location, Fig. S2). The transects started where the first marram grass vegetation was present and on the narrow and intermediate locations stretched until the foot of the foredunes (n = 21 for narrow, n = 54 for intermediate). On the wide location, the transects crossed the embryonic dune field but did not reach the foredune (n = 60). From each core the elevation was determined using RTK-GPS and the distance to the closest adult vegetation was measured. All material (shell and organic) that was left after sieving was taken to the lab to separate seeds and rhizomes from other material and count the number of seeds and rhizomes.

### Establishment field experiment

To test the establishment potential of marram grass in relation to habitat modification and beach width, a field experiment was executed from the beginning of May 2020 – March 2021. Two zones per location were selected: 1) the unmodified or beach zone between the high-water line and embryonic dune (hereafter the ‘unmodified zone’) and 2) the modified or embryonic dune zone where adult vegetation modified its environment (hereafter the ‘modified zone’). Since embryonic dunes were absent at the narrow location, here only the unmodified zone was included.

At each zone (5 in total across 3 locations as described above), we established 30 2 × 2 m plots. Each plot was assigned to one of 3 treatments ‘seed’, ‘rhizome’ or ‘control’, resulting in 10 replicates per treatment per zone and a total of 150 plots. To ensure that there was no wrack (i.e., organic material that is washed up on the beach by waves) in the plots at the start of the experiment, the top layer of the soil (± 10 cm) was raked in each plot and, if present, wrack was removed prior to sowing. An area of ± 0.5 m around the plots was cleared of vegetation. Subsequently, the seed plots were sown with marram grass seeds (1600 per plot, purchased at Jelitto perennial seeds ^®^). Since we aimed to test how the environmental conditions affected the germination, we decided to use commercially purchased seeds to minimize uncertainty of germination potential that might occur in locally harvested seeds (Del Vecchio et al. 2022). The seeds were mixed in the top 5 cm of the soil and spread across the plot. Prior to sowing, seeds were stored in dark, dry, and cold (8 °C) conditions for 4 weeks. We chose to use 1600 seeds per plot based on germination rates described by van der Putten (1990), who found that seedling numbers did not significantly increase with densities over 400 seeds m^−2^. In the rhizome plots, wrack (1600 g fresh weight per plot) was mixed with the top 5 cm of the soil. The wrack was collected in the field at the intermediate location. On average 78% of the weight of the sampled wrack consisted of rhizome parts, which had a total of approximately 3000 nodes (based on 4 subsamples). The two dominant dune grasses along the North Sea coast of Texel are marram grass and sand couch (*Elytrigia juncea*), no distinction between rhizomes of both species was made and no separation between rhizome parts and other organic material was made. Control plots were treated in the same way as experimental plots, without the addition of seeds or wrack. We assigned one of the treatments to the plots in a randomized block design. The plots were > 1 m apart and on bare soil. Shoot emergence was followed between May and October 2020. Every fortnight, shoot numbers were counted and, when present, shoot length of 10 randomly selected shoots was measured. In March 2021, the survival of individuals over winter was determined. Besides, sediment cores (ø10 cm, 20 cm depth, n = 1 per seed plot) were taken to determine retention of sown seeds in the experimental plots.

Soil moisture and salinity were determined in sediment samples (n = 5 per zone per location) from the top 5 cm of the soil twice a month from June–August. Moisture levels were determined as loss on drying (48 h at 60 °C). Water extracts were taken from the soil using 17.5 g fresh soil in 50 ml milliQ to measure soil salinity (mS/m). Sediment dynamics were measured as bed level change using a bamboo marker at one corner of each plot. Each time, the aboveground length of the bamboo marker was measured to track bed level changes. At the start and end of the experiment elevation (m MSL) was measured at each plot corner using RTK-GPS. At each location light and temperature were logged (Onset HOBO Pendant temperature/light logger) hourly between May–September on ground level, for the wide and intermediate location the logger was placed between the unmodified and modified zone and at the narrow location between the plots at the unmodified zone.

### Burial laboratory experiment

The main effect of habitat modification by marram grass is the accumulation of sand (Zarnetske et al. 2012; Reijers et al. 2019). To identify the effect of sediment accumulation on seed germination, we buried seeds (purchased at Jelitto perennial seeds ®) between 0 and 13 cm. Depths were intended to be 0, 2, 5, 8 or 13 cm, however, examination of the burial depth at the end of the experiment revealed that not all seeds ended up at the intended planting depth. For all analyses, the treatment groups were adapted to the actual burial depth which resulted in unequal group sizes (0–0.5 cm n = 400; 0.5–3 cm n = 454; 3–6 cm n = 419; 6–10 cm n = 352; 10–13 cm n = 375). Seeds were planted in germination trays (4.8*4.8*15 cm, 60 cells per tray) in untreated beach sand, which was sieved over a 1 mm mesh (collected at paal 9, Texel, the Netherlands, January 2021). Seeds were stored in dark, dry, and cold conditions (April 2020 – January 2021). To maximize germination, the seeds were wetted and kept cold (8 °C) 7 days prior to planting (van der Putten 1990). Five seeds were planted per cell and treatments were randomized per tray. Cultivation followed a day-night rhythm (12 h/12 h), simulated through changes in light intensity and temperature (day: 25 °C, night: 15 °C). Seeds were watered every other day, upon need.

The seedlings were grown for 49 days, throughout which emergence (triweekly) and shoot length (weekly) were determined. At the end of the experiment, seedlings were excavated. Subsequently, shoot (above and below sediment level) and root length were determined for a maximum of 100 seedlings per group. For a total of 176 seedlings (0–0.5 cm n = 54, 0.5–3 cm n = 78, 3–6 cm n = 50, 6–10 cm n = 13, 10–13 cm n = 2), shoots were separated from the roots, dried (70 °C, 48 h) and weighted.

### Statistical analyses

Statistical analyses were performed using the software programme R (version 4.2.1). For every test, normality of the residuals was checked and, if needed, the data were transformed using log transformation and, when transformation was ineffective, non-parametric tests were used. The reported values are mean ± se, unless indicated otherwise. *P* values lower than 0.05 were considered statistically significant.

#### Establishment field experiment

First, we determined differences in recruitment success between treatments (control, rhizomes and seeds) and zones. We defined recruitment success as the maximum number of shoots per plot over the growing season (May–October 2020). We first fitted (mixed) models (with poisson (package “lme4” (Bates et al. 2015), negative binomial (package “MASS”, (Ripley et al. 2013)), and zero-inflated (package “pscl”, (Zeileis et al. 2008)) distributions) to the data. However, neither of these models could produce a fit as the data contained only zeros in controls. Therefore, we opted for a (non-parametric) Kruskall-Wallis signed rank test combined with a pairwise Wilcoxon signed rank test with Holm correction for multiple comparisons, separating analyses between zones to achieve a balanced design (as one location lacks a zone). As a second step, we examined differences in plot success between locations and zones for the rhizome and seed treatment separately. Again, we fitted generalized linear models with poisson, negative binomial and zero-inflated distributions and selected the best fitting models based on Akaike information criterion (AIC). The best models had negative binomial distributions, which we combined with a pairwise comparison of the estimated marginal means (package “emmeans”, (Searle et al. 1980)) with a Tukey correction for multiple comparison. Finally, to test which environmental factors acted as drivers of the observed differences in recruitment success, we correlatively explored the relation between plot success and moisture, elevation and sediment dynamics using linear models. First, the change in sediment level (cm) per day per plot between every consecutive measurement was calculated. Sediment dynamics per plot were expressed as the standard deviation of the bed level change per day in cm. Subsequently, it was tested whether moisture, elevation and sediment dynamics correlated to each other using a Pearson correlation test. As (log) moisture and elevation correlated significantly (r = -0.74, *P* < 0.001, Fig. S3) and elevation was measured in a higher spatial resolution (plot level instead of every other block level), we only included elevation and sediment dynamics to test the relation between recruitment success (i.e., max shoot number) and environment using generalized linear models with a Poisson distribution. To compare the length of shoots in September (when they were on average the tallest) and survival over summer (maximum number of shoots compared with number of shoots at the end of September) between treatments (rhizome and seed) and zones we fitted linear mixed-effect models with a Gaussian distribution including location as random factor (package “lme4”, (Bates et al. 2015)). P-values were calculated in a type 3 ANOVA via Satterthwaite's degrees of freedom method (package “lmerTest”, (Kuznetsova et al. 2017)).

#### Burial laboratory experiment

Final seedling emergence and germination were compared between burial depths using a generalized linear model with binomial distribution combined with a Tukey post hoc test. To compare emergence over time between burial depths, time-to-event analyses, as described by Onofri et al. (2022), were used. A log-logistic time-to-event model was made for the different burial depths. Next, it was tested whether the model was significantly different from a reduced model using a Likelihood Ratio Test (LRT, compCDF from package drcte). Lengths and weights were compared between burial groups using ANOVA combined with Tukey post hoc tests.

## Results

### Seed and rhizome availability

Regardless of beach width (i.e., different locations) or position along the successional gradient (i.e., in modified or unmodified conditions) no seeds or rhizome fragments were found in any of the sediment cores (n = 135). Overall, cores were taken at elevations ranging from 1.5 to 7.8 m MSL and from within adult marram grass vegetation up to 5.8 m from vegetation (median 1.8 m).

### Establishment field experiment

**Fig. 2:**
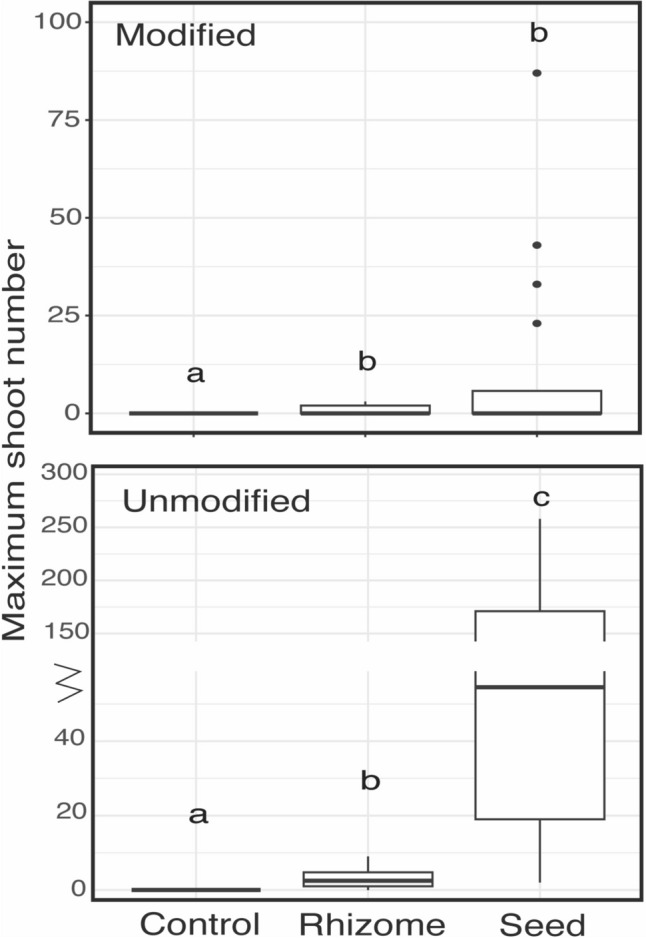
Result of the field experiment with the maximum number of shoots found per treatment in the modified zone (top) and the unmodified zone (bottom). Letters depict differences in Kruskal–Wallis combined with pairwise Wilcox signed rank test (*P* < 0.05). Horizontal lines indicate the median, box height depicts the first and third quartiles

Both in the modified and the unmodified system, more shoot emerged in the rhizome and seed plots than in the control plots, where no shoots emerged in any of the plots (Fig. 2). In the modified system, no significant difference in shoot emergence between seeds and rhizomes was found (11.5 ± 5.3 seed vs 0.9 ± 0.3 rhizome, Fig. 2). However, in the unmodified zone significantly more shoots emerged from seeds than from rhizomes (91.6 ± 15.6 seed vs 3.4 ± 0.5 rhizome, Wilcoxon signed rank, *P* < 0.001). Moreover, the difference between both zones was larger for seeds (8 times more shoots in the unmodified system, unmodified vs modified, GLM NB, z = 4.5, P < 0.001) than for rhizomes (3.8 times more shoots, unmodified vs modified, GLM NB, z = 2.7, P = 0.006). No differences in shoot emergence from rhizomes were found in the same zone between locations (Fig. S4). On the other hand, the number of seedlings (shoots originating from seed) in the unmodified zone increased by 12.5 times when the beach was wide compared to narrow (wide 190.3 ± 16.6, intermediate 69.4 ± 16.7, narrow 15.1 ± 4.4; wide vs narrow, GLM NB, z = 3.7, *P* = 0.003, Fig. S4).

**Fig. 3:**
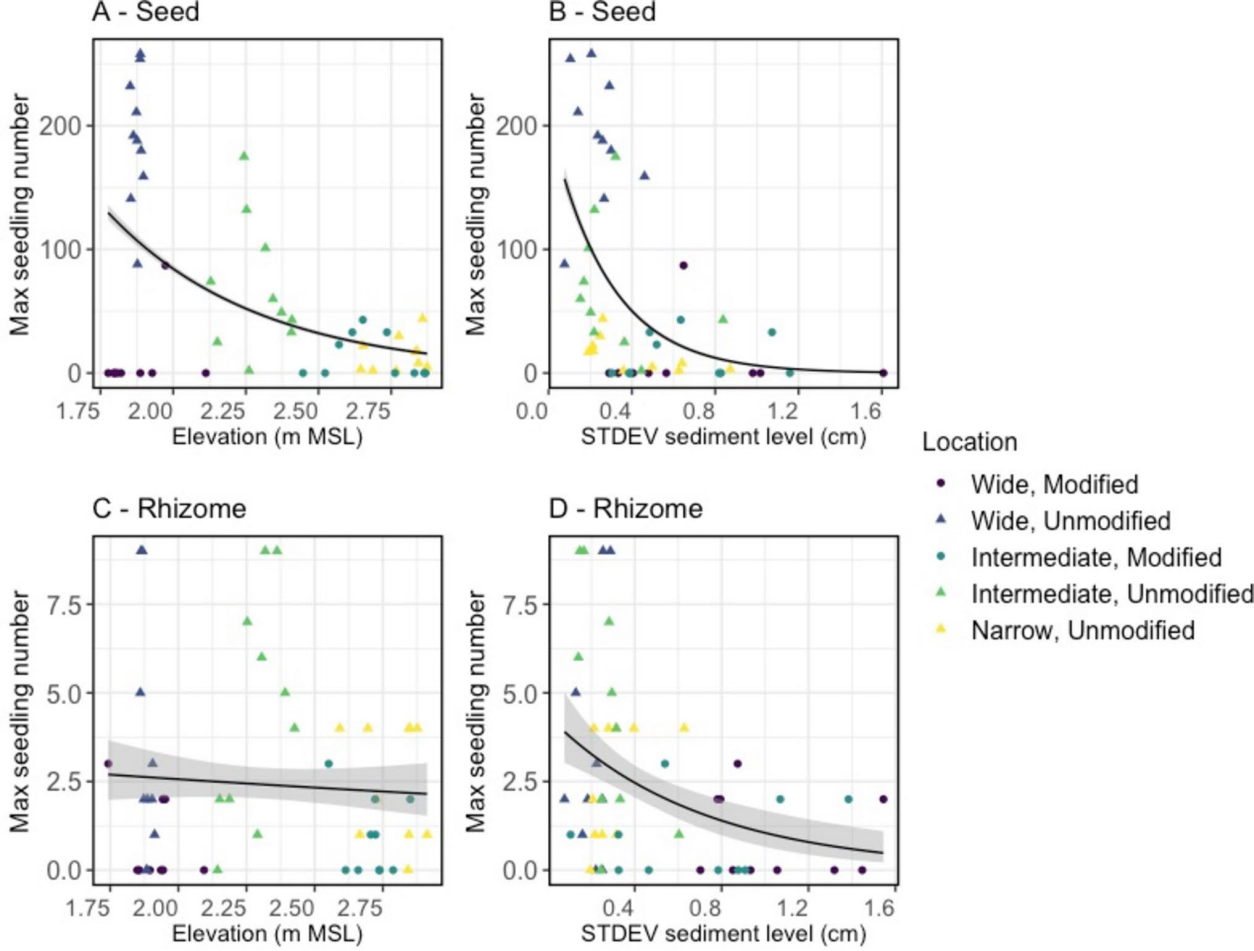
**A** & **C** Maximum seedling numbers compared to elevation (m MSL) and **B** & **D** sediment dynamics (expressed as standard deviation in sediment level (cm)), lines indicate fitted generalized linear models with Poisson distribution (in A, B & D *P* < *0.001,* and C *P* = *0.26,* regression parameters in Table S2 and S3)

Next, we tested how the plot success related to environmental conditions (i.e., elevation, sediment dynamics, soil moisture, soil salinity and temperature). Since temperatures were similar across locations, we decided to omit temperature from the comparison. Moreover, the EC in water extracts was outside the salinity range where an effect on germination could be expected (between 0 and 0.7 mS/cm, average 0.1 ± 0.03 mS/cm, compared to van Puijenbroek et al (2017a, b) and Del Vecchio et al. (2020), Table S1). Therefore, salinity was also excluded from the comparison. Besides, temperature, light and salinity levels indicated that no flooding happened during our experimental period. As elevation correlated with moisture levels (r = -0.74, *P* < 0.001, Fig. S3) we compared the number of shoots only with elevation and sediment dynamics (which were measured on a plot level, while moisture was measured every other block). Lower sediment dynamics related to a higher maximum number of shoots, both from rhizomes and seeds (GLM, *P* < 0.001, Fig. 3, Model diagnostics; Figure S5, Table S2, Table S3). Furthermore, more seedlings were found at lower elevations (GLM, *P* < 0.001, Table S3, Fig. 3) while there was no significant relation between elevation and shoot emergence from rhizomes (Table S2, Fig. 3). Generally, sediment dynamics was higher in the modified system than in the unmodified system (deviation bed level 0.74 ± 0.05 cm (modified) vs 0.31 ± 0.02 cm (unmodified), F_1,147_ = 90.96, *P* < 0.001; averages in accretion 7.5 ± 0.7 cm (modified) vs 3.0 ± 0.2 cm (unmodified) and erosion 8.4 ± 0.7 cm (modified) vs 3.4 ± 0.3 cm (unmodified)).

After the growth season, at the end of September 2020, shoots that emerged from rhizomes were on average longer than those from seedlings (15.7 ± 1.5 cm vs 11.0 ± 1.2 cm, respectively, F_1,65_ = 6.9, *P* = 0.01, Fig. S6). There were no significant differences in length between locations or zones (Fig. S6). Summer survival – i.e., the number of shoots present at the end of September compared to the maximum number of shoots per plot – was significantly higher for rhizomes than for seeds (46.1 ± 6.9% vs 12.5 ± 3.6%, respectively, F_1,65_ = 16.2, *P* < 0.001, Fig. S6). There were no significant differences in survival between locations and zones (Fig. S6). Furthermore, there were no significant correlations between sediment dynamics or elevation and survival. Almost all plants died over winter with only 22 seedlings remaining in March 2021. The remaining seedlings were all found in the same plot in the unmodified zone at the wide location.

The number of ungerminated seeds remaining in the experimental plots after winter was highest at the wide location in the unmodified system (﻿﻿29 ± 11%) followed by the intermediate location (19 ± 5%). These were higher numbers than found in the unmodified zone at the narrow location (2 ± 2%, Wilcoxon signed rank, *P* = 0.039) and in the modified system (0% (wide) and 8 ± 8% (intermediate), Wilcoxon signed rank, *P* = 0.032). Since environmental factors were not measured during winter no relation between seed retention or seedling survival and these factors could be made.

### Burial laboratory experiment

**Fig. 4:**
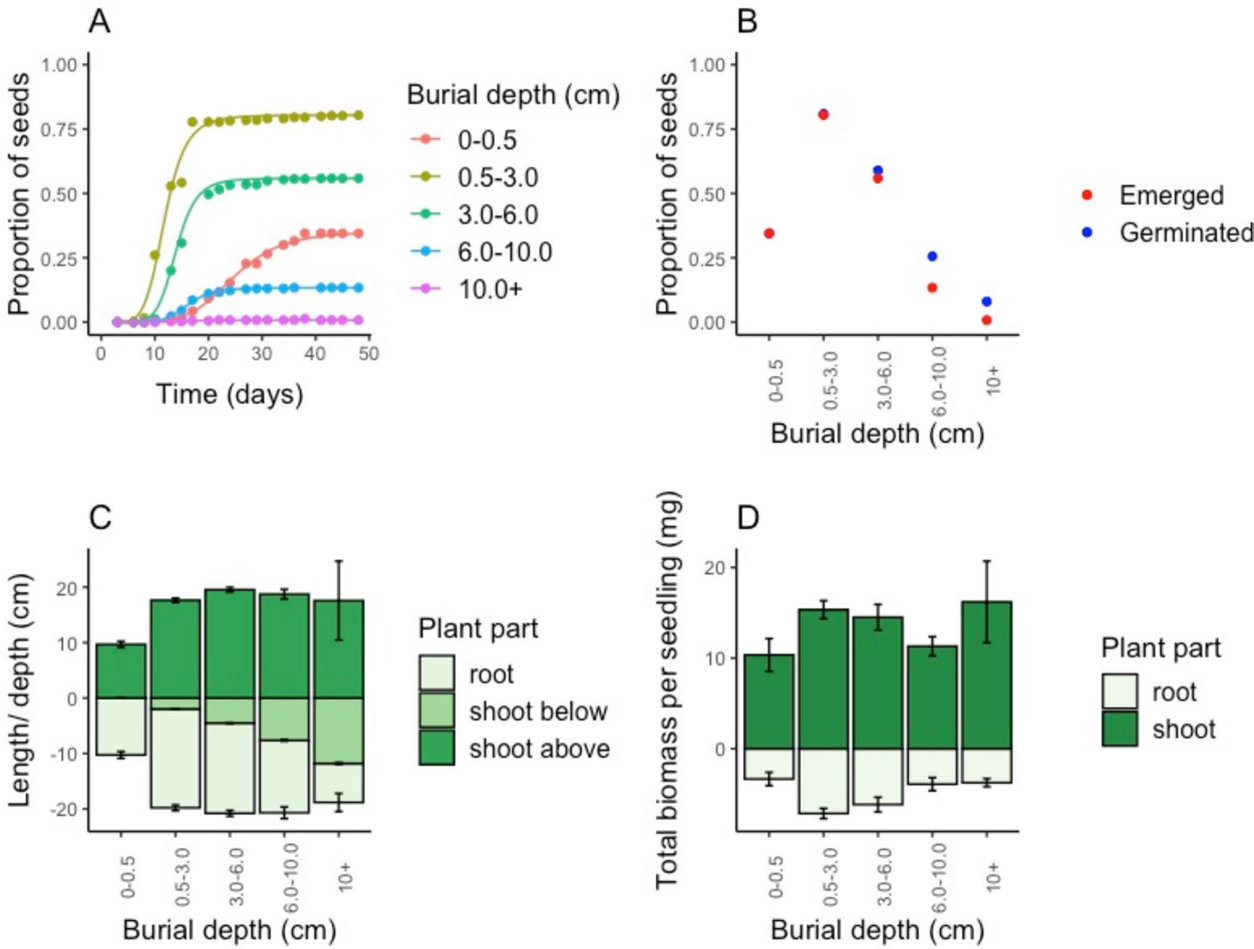
**A** The proportion of seedling emergence over time from seeds buried at different depth (indicated with different colors). The points represent the seedling counts and the lines are log-logistic time-to-event models. **B** The proportion of seeds from which seedlings emerged in red and germinated (including emerged seeds) in blue at the end of the experiment (49 days). **C** The final lengths of the shoots and roots of seedlings from different burial depths. From the shoots both above sediment and below sediment level were measured. The zero represents the sediment level and above ground parts are represented as positive values and below ground parts as negative values. **D** The total biomass per seedling with roots and shoots separated

At the end of the experiment, seeds buried between 0.5 and 3 cm had the highest emergence rate (80.4%). Seeds on top of the soil were less successful in germinating (34.5%) and more burial lead to a decrease in seedling emergence (3–6 cm 55.9%, 6–10 cm 13.3%, 10 + cm 0.8%, GLM, Tukey, *P* < 0.05, Fig. 4). Besides, with increase in burial the time to emergence increased except for the unburied seeds, which were slower to germinate (curves were unequal, LR value = 1316.21, df = 12, *P* < 0.001, Fig. 4). Similarly, growth after emergence was slower for seedlings from unburied seeds than buried seeds (which had a similar growth after emergence, Fig. S7). Next to a lower seedling emergence, also a lower germination rate (germinated but not emerged + emerged seedlings) was found with deeper burial (for all groups GLM, Tukey, P < 0.05, Fig. 4).

Seedlings originating from unburied seeds were shorter (i.e., shoot and root length combined) than the shoots buried up to 10 cm, only the emerged seedlings from over 10 cm depth were not significantly longer (ANOVA, Tukey, P < 0.05, Fig. 4). No significant differences in above and below ground length were found between the different burial depths. However, with increasing burial depth roots were relatively shorter and belowground shoot length increased (keeping the total below ground plant parts of similar length, Fig. 4). Similarly, shoot and root weights were lower for the seedlings from unburied seeds than for seeds buried up to 6 cm, but comparable to seedlings from deeper buried seeds (ANOVA, Tukey, P < 0.05, Fig. 4). Between the different burial depths again no significant differences were found. However, conversion to weight per cm tissue demonstrated that seedlings germinated from unburied seeds had a higher mass which decreased with increasing burial (Fig. S7).

## Discussion

In this study, we examined the impact of habitat modification by marram grass on recruitment of conspecifics focusing on the first two requirements for successful establishment: 1) availability of seeds and marine dispersed rhizome fragments and 2) shoot emergence. While in theory more seeds should be deposited close to vegetation (Pope 2006; McLachlan 2014), no seeds or marine dispersed rhizome fragments were found in natural conditions regardless of proximity to vegetation or beach-dune morphology. In sowed plots, seed retention over winter was higher in unmodified than in modified conditions. Moreover, burial over 3 cm significantly lowered seed germination and seedling emergence. Combined with measurements on sediment dynamics, these findings suggest that plant-induced sediment dynamics reduce seed retention as shoot emergence from seeds and rhizome fragments was severely hampered in modified conditions and was negatively related to sediment dynamics. Our findings indicate that vegetation-induced sediment dynamics negatively affect seed availability and shoot emergence from seeds and rhizome fragments, thereby hampering the establishment of conspecifics. These findings highlight the complex interaction between habitat modification and marram grass population dynamics during the early stages of dune development.

Based on the number of marram grass individuals (> 5 individuals per 100 m^2^) and estimates of seed numbers ranging between 2.000 to 30.000 seeds per individual (Salisbury 1952; Laing 1958; Lim 2012), an input of hundreds of thousands of seeds per year in the vegetated dune habitats can be expected. From these seeds, ± 78% is expected to be dispersed within a meter from the adult vegetation (Pope 2006). Yet, we found no seeds in the natural system, irrespective of proximity to adult vegetation. It is likely that plant-induced sediment dynamics reduce seed retention, which can result in seed burial with accumulation or secondary dispersal (i.e., the seeds are moved elsewhere) in erosive events. In other vegetated coastal ecosystems, such as salt marshes and seagrass beds, seed retention increases with burial depth since soil disturbance is lower at greater depth (Marion and Orth 2012; Zhu et al. 2014; Zhao et al. 2023). In coastal dunes, the same might apply, but it is likely that the dynamic upper layer is considerably larger. In summer, we found an average bed level change of 16 cm in the modified system, with erosion likely increasing in winter (van Puijenbroek et al. 2017b). Therefore, the 20 cm layer we tested might have been too shallow to confirm seed presence in more stable soil layers. It is likely that with sediment erosion, which can be caused by wind and flooding, secondary dispersal of seeds occurs. Since the dominant wind direction in the Netherlands is onshore, we expect that secondary dispersal by wind moves the seeds inland to the more stable (fore) dune areas.

While the seeds retention might increase with burial depth, we found a clear negative effect of burial on seed germination and emergence (Fig. 4). Burial over 3 cm germination rates already clearly decreased and with over 10 cm burial only 0.8% of the seeds were able to emerge. Therefore, the lack of seedlings in control plots likely results from a lack of seeds in the upper soil layer combined with a negligible germination potential from seeds that might have been buried in deeper (20 + cm) soil layers. In field conditions, we found a negative relation between sediment dynamics and shoot emergence (Fig. 3), with both accretion and erosion likely having negative effects. We did not specifically test the effect of erosion on seedlings. However, similar to other dune plants, cordgrasses and mangroves, we expect erosion to result in uprooting and desiccation of seedlings, increasing mortality (Maun 1994; Balke et al. 2013; Cao et al. 2018). We expected that higher soil moisture levels in presence of vegetation might benefit seedling establishment (Baldwin and Maun 1983), but found no significant differences in moisture levels between modified and unmodified conditions (Fig. S3). However, we did find a negative correlation between moisture and elevation (Fig. S3), and a negative relation between elevation and maximum seedling numbers (Fig. 3). Since adult marram grass vegetation entraps sediment, it increases elevation, which implicates another negative effect of habitat modification on seedling establishment. As the environmental conditions interact with each other and with adult vegetation, additional experiments with higher spatial and/or temporal resolution of measurements would be needed to disentangle specific effects of elevation, moisture, and other small-scale dynamics on seedling germination, emergence and mortality.

Similar to seeds, no marine dispersed rhizome fragments were found in our survey. Contrasting to seed input, the spread of marine dispersed rhizomes is harder to predict. Dispersal of rhizomes is dependent on storm conditions and – as rhizome bud viability is maintained after submersion in seawater up to 13 days – with longshore currents rhizomes could, in theory, be transported hundreds of kilometers (Aptekar and Rejmánek 2000; Hilton and Konlechner 2011). In our experiment, we expected a more vigorous growth from rhizomes than from seeds (Harris and Davy 1986; van der Putten 1990). Surprisingly, the number of shoots originating from rhizomes was on average 25 times lower than from seeds, while the rhizome fragments had on average 3000 nodes per plot and 1600 seeds were sown per plot. However, growth and survival of shoots over summer were significantly higher for shoots originating from rhizomes (Fig. S4). Additionally, the effect of habitat modification was less profound for rhizomes (± 4 times less growth in modified conditions) compared to seeds (± 8 times less growth in modified conditions).

We hypothesize that the difference in shoot numbers from seed and rhizomes was a result of dry conditions after the experiment started (Table S1). Although the effect of drought on marram grass rhizome fragments is unknown, for two reed species it was found that drought lowers viability of rhizome fragments (Mann et al. 2013). Similarly, we expect rhizome fragments of marram grass to be more vulnerable to desiccation than the seeds, as they had not germinated yet. The shoots that emerged from rhizomes likely had a higher resource availability than seedlings, benefiting their growth (van der Putten 1990). Increased growth enlarges their ability to deal with sediment accretion because of the reduced probability of complete burial, which is almost always fatal (Maun 1998). Moreover, rhizome fragments are expected to be less prone to erosion because seedlings rely only on their roots for anchoring while the shoots emerging from rhizomes have whole rhizome fragments as anchoring. These factors combined presumably reduced the negative effects of habitat modification on shoot survival for rhizome fragments, also resulting in a higher survival of the few shoots that emerged from rhizomes.

Marram grass is known for its high sand trapping efficiency as an adult, facilitating its own growth and survival through sediment accumulation (Zarnetske et al. 2012; Reijers et al. 2019). However, we found that these biophysical, self-facilitating feedbacks inhibit recruitment of conspecifics in early dune development. Removal of adult vegetation and associated dune forms likely plays an important role in population dynamics of marram grass, especially in embryonic dunes that are most prone to erosion (van Puijenbroek et al. 2017a; Itzkin et al. 2021). Storms can remove vegetation leaving an unmodified system where recruitment potential is higher. Additionally, storms can lead to dispersal of clonal fragments on the beach providing a source for establishment (Hesp and Martínez 2007; Hilton and Konlechner 2011). While erosion might reduce seed availability in the upper soil layers (van Regteren et al. 2019), it might also expose seeds that were previously buried in the dune body (Hilton et al. 2019). As marram grass seeds can stay viable over 21 years, seeds that were buried for a long period of time can still germinate when they resurface (Hilton et al. 2019). In addition to natural erosive events, manual removal of marram grass vegetation can have similar results. For example, recent attempts to eradicate marram grass in New Zealand (where it is an invasive species), were followed by an unexpected increase in seedlings establishment, prolonging their eradication program (Hilton et al. 2019). Overall, our results show how interactions between adult vegetation and the physical environment can inhibit recruitment of conspecifics in dune grasses and highlight the unpredictability of establishment events as these mainly occur after erosive events.

### Supplementary Information

Below is the link to the electronic supplementary material.

Supplementary file1 (PDF 2301 KB)

## Data Availability

10.25850/nioz/7b.b.xg.
