# Supplementary material for: Habitat modification by marram grass negatively affects recruitment of conspecifics
Source: Oecologia. 2024 Mar 15;204(3):705–15. doi: 10.1007/s00442-024-05525-y (PMC10980622; doi:10.1007/s00442-024-05525-y)
Supplement: Supplementary file 1 — Supplementary file1 (PDF 2301 KB) [file 442_2024_5525_MOESM1_ESM.pdf]

Supplements

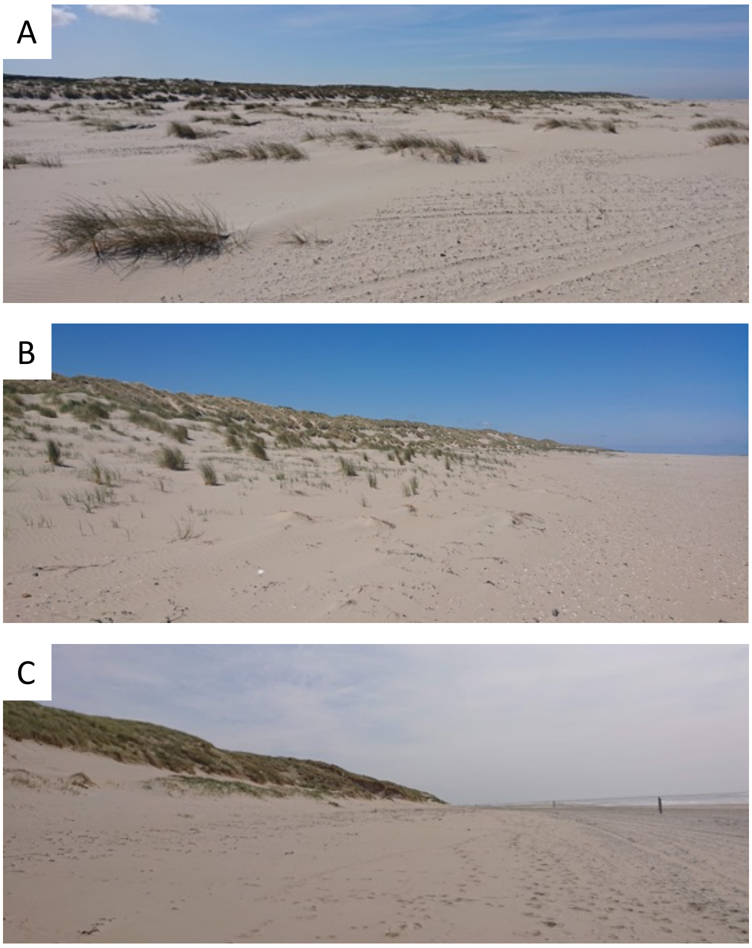

Fig S1 Impression of the three selected locations and their vegetation/successive dune stages with A) the wide location (±420m MSL-top fore dune), B) the intermediate location (±260 m MSL-top fore dune) and C) the narrow location (±140m MSL-top fore dune).

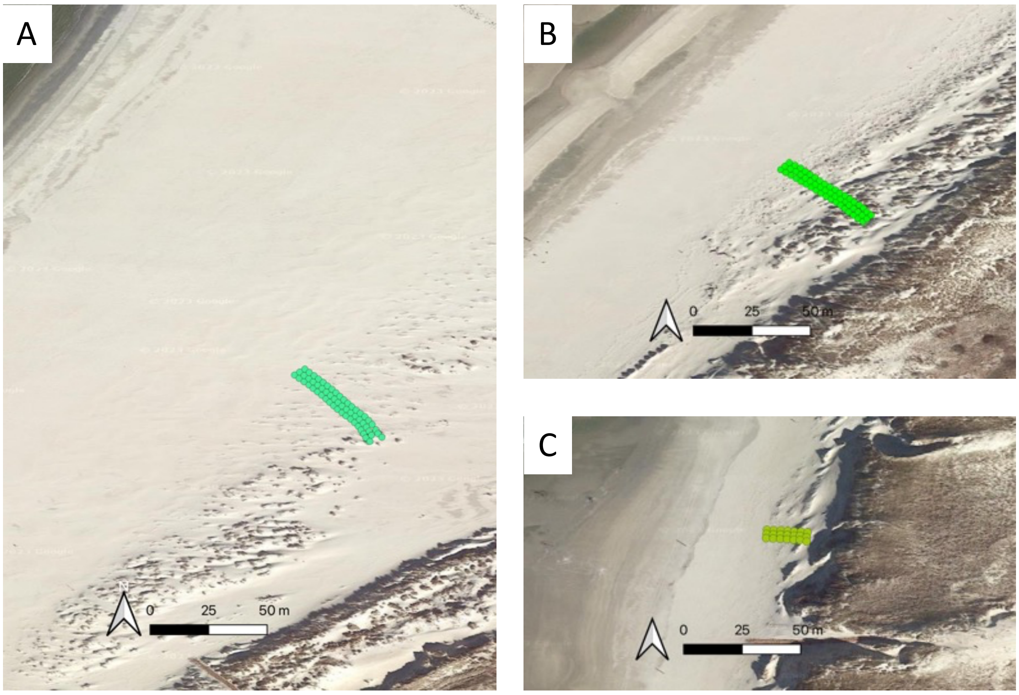

Fig S2 Places of sediment cores taken to determine seed availability, with A) the wide location, B) the intermediate location and C) the narrow location.

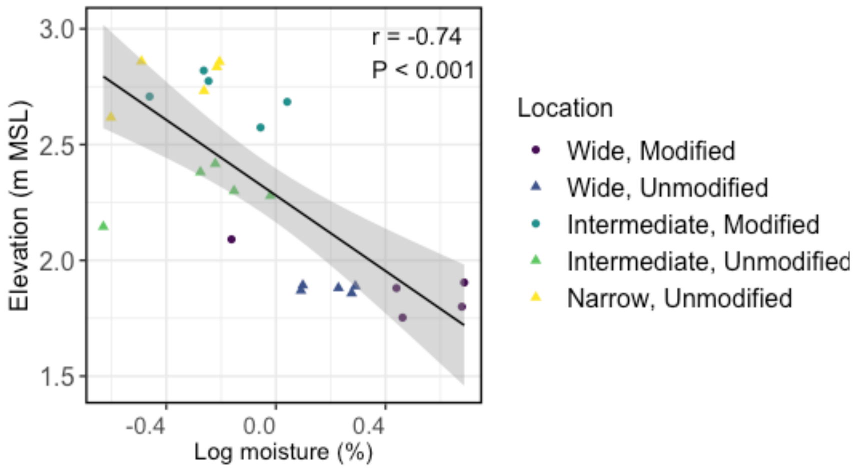

Fig S3 Relation between elevation (m MSL) and (log) moisture (%) for the field experiment. Colors indicate locations and shapes represent zones.

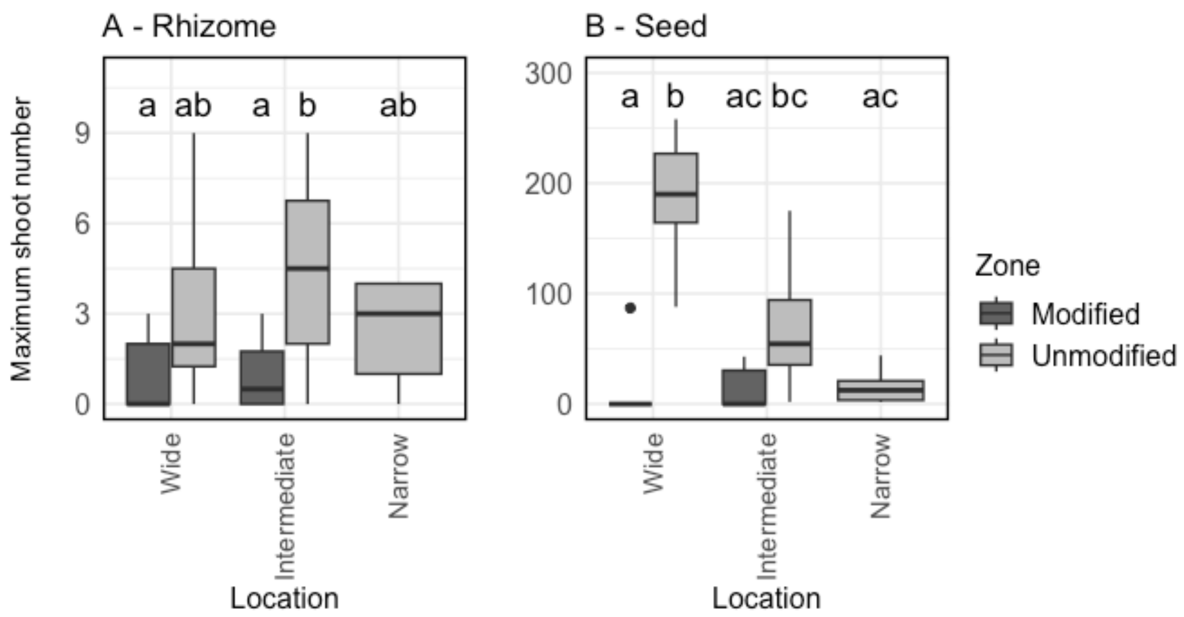

Fig S4. The maximum number of shoots per plot for rhizomes (A) and seeds (B) per location and zone (dark boxplots representing the modified zone and light boxplots representing the unmodified zone). Letters depict significant differences based on negative binomial generalized linear models combined with pairwise comparison of estimated marginal means ($P<0.05$). Horizontal lines indicate the median, box height depicts the first and third quartiles.

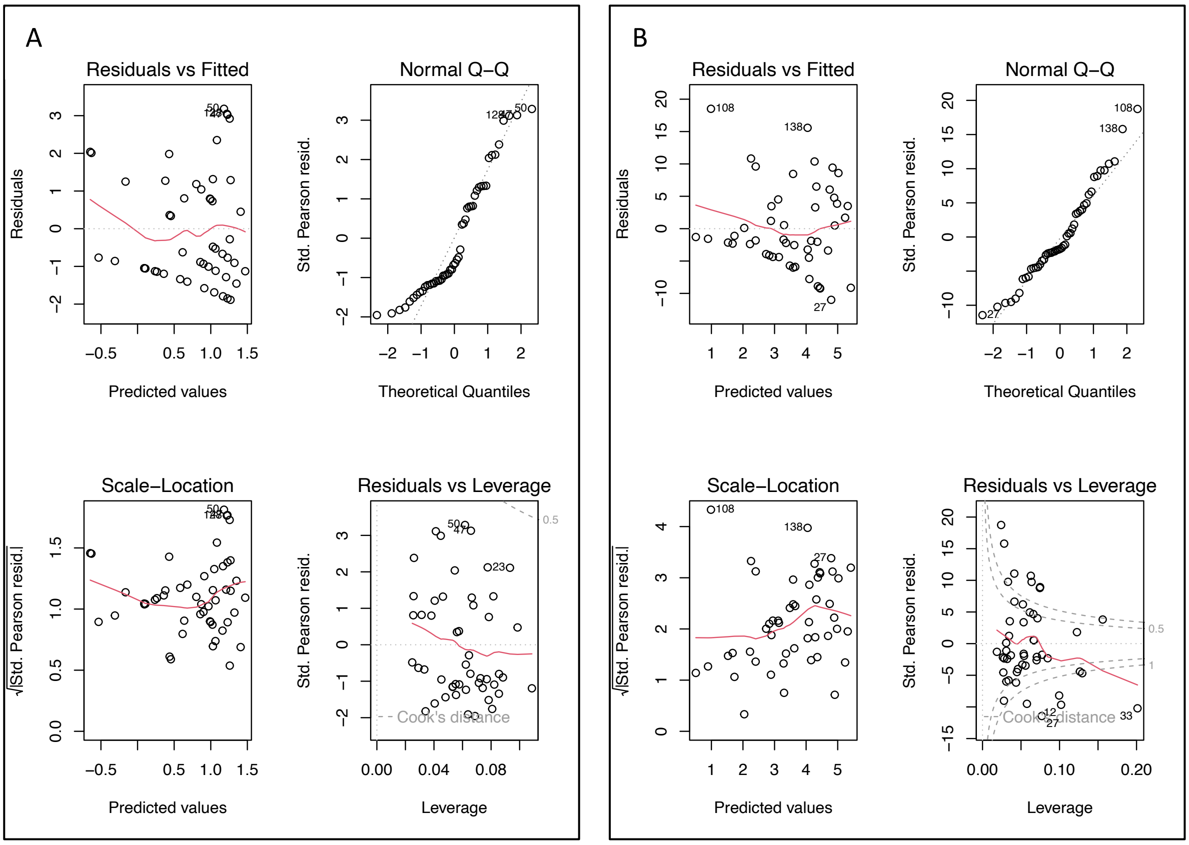

Fig S5 Model diagnostics of the GLM comparing plot success with sediment dynamics and elevation (Table S2, Table S3) for rhizomes (A) and seeds (B). Shapiro test of normality for the residuals had a p-value > 0.05.

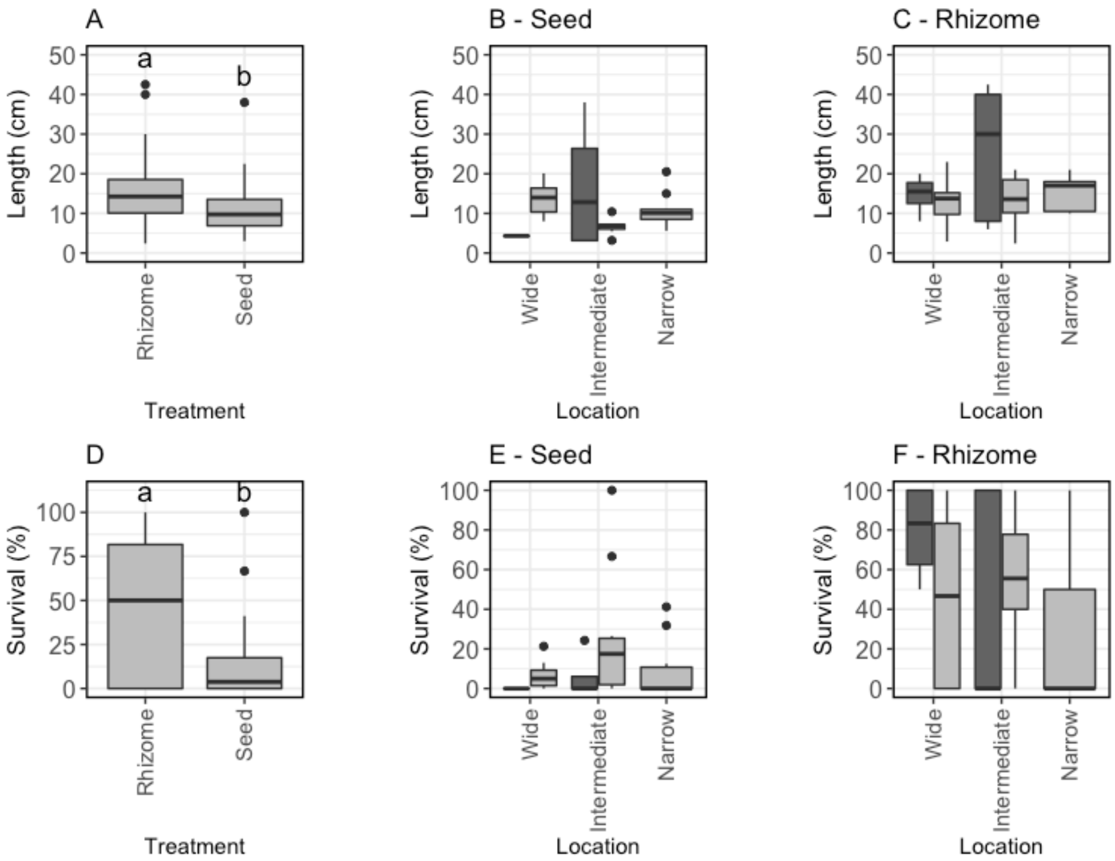

Fig S6 A), B) and C) depict shoot lengths at the end of the growth season (September 2020), A) per treatment, B) from seeds, divided per location and zone and C) from rhizome, divided per location and zone. Lengths were averaged per plot (plots with shoots per treatment=35, n=10 shoots per plot). D), E) and F) depict survival of shoots over summer (i.e., maximum number of shoots divided by the number at the end of the growth season), D) per treatment, E) from seed, divided per location and zone and F) from rhizome, divided per location and zone. Letters depict significant differences using Kruskal Wallis test (P<0.05). Horizontal lines indicate the median, box height depicts the first and third quartiles.

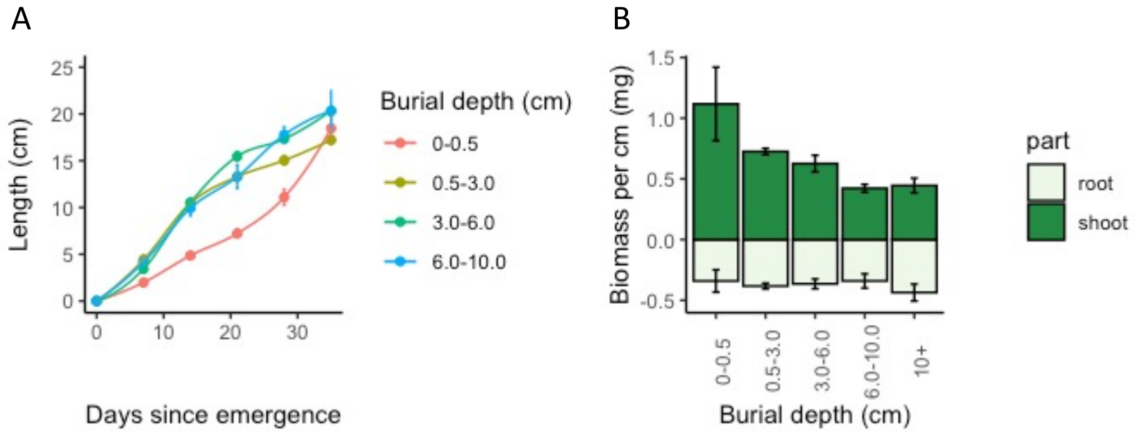

Fig S7 A) Seedling growth after emergence for seedlings originating from different burial depths (indicated by different colors). Seedlings that turned out to be buried deeper than 10cm were not individually followed over time, therefore that group is not included. B) Biomass per cm for seedlings from seeds buried at different depths separated between shoot and root biomass.

Table S1: Environmental data. Max daily temperature measured with HOBO onset temperature loggers. Soil salinity was measured in sediment samples (see Methods) and rain data from the Cocksdorp (closest measuring point) were downloaded from the Royal Netherlands Meteorological Institute (https://www.knmi.nl/nederland-nu/klimatologie/monv/reeksen)

	**Wide**	**Intermediate**	**Narrow**
Max daily temperature at sediment level (May – August)	29.2±0.5	30.4±0.6	30.8±0.7
Average soil salinity (EC in soil solution, mS/cm)	**0.2±0.04**	**0.02±0.005**	**0.03±0.005**
Rain (mm, May 2020)	**12.8**		
30-year average rain (mm, May)	**44.3±4.1**		

Table S2 Estimated regression parameters, standard errors, z-values and *P*-values for the Poisson generalized linear model comparing shoot numbers originating from rhizomes with sediment dynamics (Deviation sediment height) and elevation.

	Estimate	*Std. error*	*Z value*	*P value*
Intercept	2.10	0.57	3.69	<0.001
Deviation sediment height (cm)	-0.27	0.23	-1.13	0.26
Elevation m MWL	-1.42	0.33	-4.26	<0.001

Table S3 Estimated regression parameters, standard errors, z-values and *P*-values for the Poisson generalized linear model comparing seedling numbers (i.e., shoots originated from seeds) with sediment dynamics (Deviation sediment height) and elevation.

	Estimate	*Std. error*	*Z value*	*P value*
Intercept	9.02	0.13	68.76	<0.001
Deviation sediment height (cm)	-2.90	0.11	-26.57	<0.001
Elevation m MWL	-1.80	0.06	-28.58	<0.001
